# Understanding marital violence: a study in grounded theory*

**DOI:** 10.1590/1518-8345.3116.3185

**Published:** 2019-10-07

**Authors:** Jordana Brock Carneiro, Nadirlene Pereira Gomes, Luana Moura Campos, Andrey Ferreira da Silva, Kamylla Santos da Cunha, Dália Maria De Sousa Conceição Da Costa

**Affiliations:** 1 Universidade Federal da Bahia, Escola de Enfermagem, Salvador, BA, Brazil.; 2 Scholarship holder at Coordenação de Aperfeiçoamento de Pessoal de Nível Superior (CAPES), Brazil.; 3 Scholarship holder at Fundação de Amparo à Pesquisa do Estado da Bahia (FAPESB), Brazil.; 4 Universidade Federal de Santa Catarina, Departamento de Enfermagem, Florianópolis, SC, Brazil.; 5 Scholarship holder at Conselho Nacional de Desenvolvimento Científico e Tecnológico (CNPq), Brazil.; 6 Universidade de Lisboa, Instituto Superior de Ciências Sociais e Políticas, Lisboa, Portugal.

**Keywords:** Domestic Violence, Intimate Partner Violence, Family Conflict, Gender Relations, Grounded Theory, Social Support, Violência Contra a Mulher, Violência por Parceiro Íntimo, Conflito Familiar, Gênero, Teoria Fundamentada, Rede Social, Violencia Contra la Mujer, Violencia de Pareja, Conflicto Familiar, Identidad de Género, Teoría Fundamentada, Red Social

## Abstract

**Objective:**

to understand the phenomenon of marital violence based on the experience of women in judicial process and network professionals.

**Method:**

a qualitative study, with theoretical-methodological support in grounded theory. Data collection took place in two regional Courts for Peace in the Home in a municipality of the Brazilian Northeast. Interviews were conducted with 38 participants, who composed two sample groups: women in situations of violence and network professionals.

**Results:**

the understanding of marital violence emerged for the phenomenon “Experiencing marital violence as a progressive and cyclical process, with repercussions for health and implications for social relations”.

**Conclusion:**

in recognizing marital violence as a recurring problem in the life of women, with implications for their own health and that of their children, the study points to the relevance of coping strategies based on institutional and social support.

## Abstract (pt)

**Objetivo:**

compreender o fenômeno da violência conjugal a partir da experiência de mulheres em processo judicial e de profissionais da rede de atenção a mulheres em situação de violência.

**Método:**

estudo qualitativo, com aporte teórico-metodológico na grounded theory. A coleta de dados ocorreu em duas varas de Justiça pela Paz em Casa de um município do Nordeste brasileiro. Realizou-se entrevista com 38 participantes, que compuseram dois grupos amostrais: mulheres em situação de violência e profissionais da rede.

**Resultados:**

a compreensão da violência conjugal despontou para o fenômeno “Vivenciando a violência conjugal como um processo progressivo e cíclico, com repercussões para a saúde e implicações para as relações sociais”.

**Conclusão:**

ao reconhecer a violência conjugal enquanto um problema recorrente na vida da mulher com implicações para sua saúde e de seus filhos, o estudo aponta para a relevância de estratégias de enfrentamento do fenômeno a partir do suporte institucional e social.

## Abstract (es)

**Objetivo:**

comprender el fenómeno de la violencia conyugal desde la experiencia de mujeres en proceso judicial y de profesionales de la red.

**Método:**

estudio cualitativo, con aporte teórico-metodológico en la grounded theory. La recogida de datos ocurrió en dos Jurisdicciones de Justicia por la Paz en Casa de un municipio del noreste brasileño. Se realizó encuesta con 38 participantes, que conformaron dos grupos de muestra: las mujeres en situación de violencia y los profesionales de la Red.

**Resultados:**

la comprensión de la violencia conyugal emergió el fenómeno “Vivenciando la violencia conyugal como un proceso progresivo y cíclico, con repercusiones para la salud e implicaciones para las relaciones sociales”.

**Conclusión:**

al reconocer la violencia conyugal como un problema recurrente en la vida de la mujer con implicaciones para su salud y la de sus hijos, el estudio indica para la relevancia de estrategias de enfrentamiento del fenómeno desde el soporte institucional y social.

## Introduction

With roots in gender inequality, marital violence has been experienced for many years, constituting itself as a public health problem. The challenge to intervene in this problem requires a better understanding of the interactional processes that pervade this phenomenon.

Women’s vulnerability to experiencing violence is anchored in the socially constructed view of man as domineering, strong, virile and insensitive, and of women as delicate, faithful and submissive^(^ 1 ^)^. The different attributes, taken as inherent in men or women, derive from the social-historical construction that determines gender inequality, legitimating female inferiority and male social and sexual superiority^(^ 2 ^)^. These asymmetries favor abusive intimate relationships, leaving women prone to violence in conjugality^(^ 3 ^)^, as well as to remaining for years in this relationship^(^ 4 ^)^.

The prolonged nature of this experience, as well as the high prevalence of the harm, makes it an important public health problem. In Rwanda, a survey of 1,821 women revealed that 68% of these women were victims of intimate partner violence^(^ 5 ^)^. This reality was also evidenced in our country, as a study that investigated the distribution of conjugal violence across Brazilian states found that reported cases almost tripled between 2009 and 2014, with a significant increase in cases in the Southeast, South and Midwest^(^ 6 ^)^.

The problem is further compounded by the expenses related to health demands generated by the repercussions of this harm. In 2004 alone, Brazilian health services spent 90.2 billion *reais* on prevention, treatment and rehabilitation of women victims of marital violence^(^ 7 ^)^. This cost, coupled with the expenses with social and legal-police services, end up compromising the economic productivity of nations. In this regard, the United Nations estimates that partner violence-related costs in the US are nearly six billion dollars and in England and Wales approximately $33billion^(^ 8 ^)^.

Despite these burdens, many cases of marital violence have not been identified in the health services. A national survey carried out with professionals who work in family health teams in Florianópolis, Santa Catarina, Brazil, draws attention to the difficulty of recognizing the problem in the Family Health Strategy (ESF) scenario, which is a fundamental preparation, especially for the nursing professional who, besides integrating the minimum team, has been in charge of the management of services^(^ 9 ^)^. It is worth emphasizing that the performance of health professionals is a key element in the detection of cases of violence and in the promotion of women’s health and well-being, where the referral to the coping network is an essential step for prevention of the damage resulting from this experience^(^ 10 ^)^.

Considering the relevance of professional preparation for the suspicion of ongoing marital violence as a cause associated with demands in health services, the theoretical deepening regarding the perception of the phenomenon is necessary not only by women who have experienced the problem but also by professionals of the services that integrate the network of attention to women in situation of violence. Believing that such an understanding is essential for the definition of strategies for prevention and coping with this problem, the following objective was outlined: Understanding the phenomenon of martial violence from the experience of women in judicial process and network professionals.

## Method

A qualitative research study linked to the original project “Re-education of men and women involved in criminal prosecution: a strategy to fight marital violence”, which aimed to develop social technology for prevention and coping with conjugal and gender violence. This project was funded by the State of Bahia Research Foundation (FAPESB) in partnership with the Public Security Secretariat of the State of Bahia (SSP/BA).

The study scenario comprised two regional Courts for Peace at Home, located in the city of Salvador, Bahia, Brazil. These, created in 2008 and 2014, comply with the recommendation of Law 11.340/2006, which calls for the creation of special regional courts to conduct cases of domestic and family violence against women and were chosen because they are responsible for prosecuting marital violence cases.

For the development of this study, the Straussian strand of grounded theory was used as a theoretical-methodological basis. This method offers greater understanding about a particular phenomenon through people’s actions and interactions in a given context. Its objective is not to verify a preconceived theory, but to generate a theory with rigor to social research anchored in the constant comparative analysis of data^(^ 11 ^)^. It should be noted that grounded theory enables researchers to initiate research with an open mind, attentive to the answers of the initial participants who were chosen intentionally, exploring the richness and diversity of their meanings and experiences to achieve the goal outlined.

Thus, the theoretical sampling of the study can be obtained by means of sample groups, with equal or different participants, but with relevant experiences to the phenomenon under investigation^(^ 11 ^)^ . In this sense, we purposely chose for the first group women who met the following inclusion criteria: being in legal process for conjugal violence in the regional Courts and being over 18 years of age. Women whose emotional and psychological state was contraindicated by the Courts’ psychosocial service were excluded.

Considering said criteria, women were invited by the psychologist and/or social worker of the Courts to integrate Discussion Groups (DG), made viable by the original project, whose proposal was to promote a reflective space for women with a history of conjugal violence in order to contribute to their strengthening. This strategy was used as a process of approaching the participants, since violence is understood as an intimate problem and thus difficult to approach.

Semi-structured interviews with study participants were scheduled during the DG and subsequently conducted by a master’s student and a PhD student in nursing. It is important to mention that the researchers followed all the meetings and, because they are part of Grupo Vid@, they are highly experienced in activities with women in situations of violence. In addition, they conducted pilot interviews for testing the form, which had two parts: the first, with closed questions, addressed sociodemographic characteristics (age, marital status, family income, religion, schooling, number of children) and the second, with open questions, addressed the following guiding question: “Tell me about your experience of marital violence.” Based on their answers, new questions emerged by initiating a conversation aimed at reaching the depth of data in its properties, dimensions and research progress.

The single and individualized interviews were conducted between May and September 2015 at the participants’ place of choice, i.e., either at their homes or at a room provided by the state school where the DGs were held. Meetings lasted in average one hour and 25 minutes, and the interviews were fully recorded and transcribed for subsequent analysis.

Over the process of continual collection and analysis of data from the first sample group, the following hypothesis was formulated: “Women who experience marital violence seek professional support in the Network of Attention to Women in Situation of Violence.” This hypothesis directed the continuity of data collection for the second sample group, composed of professionals of the services mentioned by the women. The interviews with the participants of the second sample group lasted an average of 55 minutes and were previously scheduled by telephone and performed individually in rooms provided by the different services in which they worked (Regional Courts of Violence, Public Prosecutor’s Office and Public Defender’s Office of the State of Bahia) from November 2015 to January 2016. The dialogue for this group started from the following guiding question: How does the service to women in situation of marital violence occur in the Network? Other issues were implemented along the conversation to achieve depth of data.

From the repetition of information and absence of new relevant data to be explored to deepen the research objective and understand the phenomenon, theoretical saturation of the data was reached^(^ 11 ^)^. During the process of constant comparative analysis of data and concept emergence, memos and diagrams were produced concerning the researcher’s reflections on data, which contribute to illustrate ideas and codes that aid theory development. The NVIVO^®^10 software was used to organize the data during the period of comparative analysis and coding.

Data coding occurred in three interdependent stages: open, axial and selective coding^(^ 11 ^)^. In open coding, concepts were identified and grouped into categories according to their similarities. Then, in axial coding, categories and subcategories were related in order to obtain a more detailed explanation by means of a systematic analytical comparative process guided by the five-component paradigmatic model of the Straussian strand of grounded theory: *context* - where and in what circumstances the phenomenon occurs; *causal condition* - happenings, events or incidents leading to the occurrence or development of a phenomenon, circumstances or situations where the phenomenon is incorporated; *intervening condition* - which changes the impact of causal conditions on the phenomenon and may facilitate, hinder or even restrict the strategies adopted; *strategies* - refer to the actions developed to achieve the phenomenon; and *consequences -* results of actions-interactions concerning a given phenomenon^(^ 11 ^)^. Finally, in the selective coding stage, the relations, associations and interactions between these five categories were interconnected around a central category, giving rise to the study phenomenon.

The theoretical model proper was validated by five researchers with expertise in the method and also with the research participants. In the validation, we sought to verify methodological coherence, thematic adherence and possibilities of theory abstraction. It should be noted that this study was approved by the Ethics Committee of Research with Human Beings of the Federal University of Bahia under opinion nº 039699/2014 and Certificate of Presentation for Ethical Appraisal nº 877.905/2014. Participants expressed their consent by signing a Free and Informed Consent Form. To protect participants’ identities, women and professionals were identified by the letters “W” and “P”, respectively, followed by an Arabic numeral indicating interview order.

## Results

The theoretical sample of the study comprised 38 participants, divided into two sample groups. The first group was composed of 29 women aged from 25 to 71 years (mean = 41 years). All of them lived in peripheral neighborhoods, and 65% declared themselves as black or brown. Regarding schooling, although 62% of the participants completed high school or college and a representative percentage had some paid activity (76%), family income was less than two minimum wages (79%). Concerning marital status, the majority was married or lived in common law marriage (62%) and had children (86%).

The second sample group consisted of nine professionals who worked in services of Women in Situation of Violence Assistance Network (Regional Courts of Violence, Public Prosecutor’s Office and Public Defender’s Office). They were: a social worker, a psychologist, a prosecutor, two public defenders, two conciliators and two female judges. Although all the interviewees in this group were women, such homogeneity in the gender composition of the sample was not intentional, resulting from the characteristics of the trend of feminization in this professional area.

The findings of the study converge to the meaning of experiencing marital violence by women in criminal prosecution revealed by the phenomenon “Experiencing conjugal violence as a progressive and cyclical process, with repercussions for health and implications for social relations.” The understanding of such meaning emerges from five components of the paradigmatic model.

The data revealed a context of conjugal violence experienced in a cyclical way through the category “Experiencing conjugal violence in a cyclical way”. In it, women experience the phenomenon expressed in patrimonial, psychological, moral, sexual and physical forms. This experience takes place progressively, beginning with more veiled expressions of violence, such as jealousy, control, humiliations and social isolation of women, and progressively evolves into increasingly explicit expressions using physical force, culminating in a more serious event, such as hangings and battering. Following this apex event, the offender is repentant and promises changes in behavior. This cycle tends to recur at increasingly shorter intervals and with increasingly severe apex events*. He said he wanted me just for him; he would not let me see my family and I thought it was because he loved me* [...]. *Eventually he began to curse me and humiliate me.* [...] *he has already put me and my son out of the house.* [...] *he would rape me, hang me, punch me in the eye, the head* [...]. *After the assaults, he apologized, promised that it would not happen any more, and I believed and forgave him* (W1, 29); *He would not let me work, study, or receive my friends.* [...] *he began to curse me, humiliate me, accuse me of treason, beat me up. Then he came with affection, he really seemed to be sorry. I thought our relationship had a chance, but it got worse and worse* [...] (W11, 31). [...] *she deludes herself that she will be able to change her partner. Only after making several attempts does she come for help* (P1).

The consequences component, which has emerged in the category “Pointing out repercussions of conjugal violence for women and children”, points out that experiencing conjugal violence in intimate relationships brings repercussions to the lives and health of women and their children, for example, by compromising children’s school performance, who also tend to reproduce violent behavior as adults. *He hit me so hard that I went to hospital with bleeding.* [...] *I have already had gastritis, my psychological was shaken, I did not shower, did not want to talk to people. Because of this situation, my son has become an aggressive child and is seriously struggling in school* (M14, 49); *Many children who witness or are victims of violence become depressed, aggressive and have difficulty in relating* (P7).

From the category “Unveiling the Transgenerationality of Violence”, the data revealed the situations in which the phenomenon was generated and how it is incorporated into the lives of couples, since both women and their aggressors witnessed and experienced violence in their families of origin, reproducing it in their relationships. *His parents would beat him and he would say that when he grew up he would be a different father, but he also assaulted our son* [...]. *Today I can see that my marriage was the same as my parents’* (M5, 57); *My ex-husband was raised in a very violent house. In my childhood, I saw my father fighting with my mother as well, but she always complied with what he said.* (W23, 38); *It’s a cultural thing. This way of relating goes from father to son and mothers also pass to daughters that it’s normal because it was so with her* (P3).

As regards the intervening condition, which acts on the central phenomenon, increasing its complexity, the study revealed in the category “Missing family support” that, during the experience of violence, family support is non-existent. This condition weakens the woman’s decision making to break up the relationship. *My brothers knew about the violence, but they did not get involved. I did not have any support to be able to fight* (W6, 38); *Had I had adequate support, I’d already have broken up with him a long time ago. My mother used to say that men were like that; that I needed to know how to deal with my marriage and keep my husband; that should I separate, the next one could be the same or worse* (W28, 37); *Women who do not have family support seldom get out of the relationship or report* (P5).

The strategies component of the paradigmatic model, shown in the category “Seeking institutional and social support”, evidences women’s search for institutional and social support, represented by family support and participation in support groups, which contribute to coping with marital violence. *At the women’s police station, they explained to me about the progress of the case, they referred me to the Public Defender’s Office and the regional Court. They also arranged for me to participate in women’s workshops. Through this, I managed to make plans and go on with my life* (M4, 32); [...] *They issued the protective measure and since then he did not look for me any more.* [...] *my son also stood by me, hosted me in his home, helped me financially.* (M3, 71 years); *At the police station they should be directed and referred to the Reference Center or shelter homes.* [...] *when she has support from family, friends, and church she strengthens herself to break the relationship* (P2).

## Discussion

As a method that aims to understand how social beings deal with their experiences^(^ 11 ^)^, grounded theory allowed us to point out that women experience a daily cycle of conjugal violence in a cyclical way. It is important to mention that this reality, present in all spheres of society, affects women in situation of social vulnerability^(^ 12 ^)^, represented in our study fro being black, low income and living in peripheral locations, these last two characteristics also make women vulnerable in developed countries such as Canada and Spain^(^ 13 ^-^ 14 ^)^. The repetitive context, initially part of more veiled expressions of violence, such as swearing, deprecation, humiliation, defamation, which represent the phase of accumulation of tension. Such events progressively evolve to culminate in an episode of greater gravity, which is usually revealed in physical form. A study in Iran also points to the cyclical and progressive character of conjugal violence, which begins in a milder way, commonly expressed in psychological form^(^ 15 ^)^. This event gradually intensifies throughout the relationship, culminating in an episode of greater severity, which usually manifests itself in physical form^(^ 15 ^)^.

Even after experiencing an acute event of violence, it is common for women to resume their relationship, as the data show. This condition expresses the “honeymoon” phase, in which the man shows repentance and the woman grants the pardon, action that is linked to the expectation of a behavioral change in the former^(^ 16 ^)^. In this respect, research carried out in Iran and Portugal also showed women’s permanence in the conjugal relationship, whose estimated time is over ten years, as she believes in spouse change^(^ 17 ^)^. The belief in spouse change is often tied to convictions about inseparability of marriage, overvaluation of the father figure in child rearing, and the social stigma of divorce^(^ 17 ^)^.

Regardless of the reasons for staying in a violence-ridden marital relationship, the study reveals that experiencing the harm eventually affects women’s physical and emotional health. As an example, we can mention research that emphasizes different repercussions of conjugal violence on the physical integrity of women, such as cuts, lacerations, bruises, fractures, traumatic brain injury and polytrauma^(^ 4 ^)^. Regarding mental impairment, the study points to marital violence predisposing to such mental illnesses as depression, acute stress, anxiety, and even bipolar disorder and schizophrenia^(^ 18 ^)^. These manifestations point to the need for integral actions aimed at reestablishing women’s physical and psychological health.

In addition to health damage, a conjugal daily life permeated by oppressive acts and control impacts on women’s social relations. This is because the restriction of female liberty, often mistaken for a manifestation of love and care, forcefully deprives a woman from family and social life. As in our study, a survey conducted in the US with 29 women in situations of marital violence signaled their difficulty in establishing affective bonds precisely because, in this type of relationship, it is common for the spouse to control all aspects of the woman’s life, and in many cases to forbid her from engaging in any professional activity^(^ 19 ^)^.

This male domination over women has implications for their professional lives, thus impacting on women’s ability to provide for themselves and their offspring. This situation often occurs by the man favoring motherhood and marriage and women giving up training for the labor market and failing to engage in paid activities, as pointed out in a Spanish study of women seen in primary health care^(^ 14 ^)^, who become increasingly dependent on their partners and the marital relationship.

The damage resulting from conjugal violence is not restricted to the consequences in women’s health, since speeches point to implications on children, in the form of social isolation, low school performance and aggressive behavior. National and international studies have also indicated the extent of these repercussions for children^(^ 20 ^-^ 21 ^)^. In addition, witnessing harm between parents and/or being a victim in childhood and adolescence is significantly related to all types of violence suffered by women and manifested by men^(^ 22 ^-^ 23 ^)^. It reveals the transgenerational character of the offense, pointed out in our study as a causal condition for experiencing violence in conjugality, since it leads to the development of the phenomenon.

As a condition that interferes with the phenomenon, the study pointed to the lack of family support as an event that weakens the woman in deciding to end the conjugal relationship. An international study of married women in Afghanistan has revealed that, because women believe that it is their job to maintain harmony at home, the family tends to hold her accountable for the violence suffered by supporting the man, who uses aggressive means to demand behaviors and standards understood as normal^(^ 24 ^)^. As in our study, other studies show the maintenance of female submission in romantic relationships anchored in the belief of women’s inferiority to men, a cultural value communicated within family relationships, which hinders family support to break up with the relationship^(^ 24 ^-^ 26 ^)^. This construct favors women’s acceptance of violent attitudes by the spouse from the understanding that they should and/or deserve to be subjected to violent acts in an intimate relationship, according to a study carried out in Cape Town, South Africa^(^ 27 ^)^.

However, women’s experience of marital violence shows that family support, if any, has proven to be a powerful support strategy that is central to women’s strengthening so as to be able to nullify the experienced violence^(^ 28 ^)^. It can be inferred, therefore, that a cozy family space offers enough security to reach an internal transformation in the woman, removing her from a state of inertia and submission, which favors a break with the scene of violence^(^ 24 ^)^.

In addition to emotional support, the data point to the importance of financial support from family members in the decision to break with the abusive relationship. Although most of the interviewees reported earning less than two minimum wages, this condition does not guarantee them a favorable financial structure. This question can be justified by the fact that, in a context of conjugal violence, men tend to subtract/control women’s money^(^ 29 ^)^. Another circumstance arises from marital separation, where the ex-spouse does not provide for the family and the woman has to take alone all the financial responsibilities, both of the home and the children. A study carried out with women in three provinces of Ecuador showed that it is common, after separation, that fathers stop providing for their children financially^(^ 30 ^)^, leaving all expenses to women.

In addition to family support, the study outlines other strategies for encouraging women to leave the violent setting, such as support from friends, religious institutions and discussion groups. These have already been considered crucial in the creation of mechanisms for decision through complaint and, mainly, by contributing with the necessary emotional support for establishing their psychological health^(^ 28 ^)^. Research conducted in the USA showed that the network of neighbors, family and friends acts in a protective way against mental illness and suicidal behavior^(^ 31 ^)^, indicating that such support has a positive influence in preventing social isolation. Another study carried out in six European capitals showed that higher levels of social support were associated with lower frequency of victimization by domestic violence^(^ 32 ^)^.

Concerning these women’s participation in discussion groups, our study portrays them as a possibility of social support, since these spaces can contribute to the re-signification of lived experiences, with consequent creation of new perspectives for the future, as indicated by the participants. This reality was shared by Brazilian research with women taking part in a Non-Governmental Organization (NGO) created to face domestic violence, which showed group support as an important element that allows women to better understand unequal gender relations, making them recognize themselves in a situation of violence, which promotes reflection on their lives and encourages them to make decisions^(^ 28 ^)^.

This model of collective group action can still be incorporated by institutions, such as police stations and regional Courts, which were also flagged as a strategy that favors female empowerment and perhaps a break in the cycle of violence. Such scope ranges from instructing these women about the progress of the legal-police process to doing the necessary referrals, which include police, legal, psychosocial and health support. Therefore, these services should be integrated into a network in order to provide women in situations of violence with the necessary protection according to their demands and, therefore, they must act in an articulated manner^(^ 20 ^)^. These scenarios, which make up the network of assistance to women in situations of violence, are important because they encourage women to leave the situation of vulnerability involving domestic violence^(^ 33 ^)^ , by paying attention to their needs in an effective way^(^ 34 ^)^.

Although the findings corroborate the complexity of domestic violence, according to production of the national and international knowledge, the discovery of an explanatory conceptual model of the phenomenon investigated by the relationship and interaction of conceptual categories and between the components of the paradigmatic model^(^ 35 ^)^ allows to understand the complexity of the aspects that permeate the experience of violence within the intimate relationship, presented here in an articulated and non-fragmented way. By allowing a deeper understanding of the phenomenon, it is believed that the emergence of this substantive theory allows the development of a management model capable of directing strategies for prevention and coping with the problem.

Given the relevance of services for women in situations of conjugal violence, especially in the process of action and interaction with them, it is important to emphasize the importance of professional preparation to deal with this complex problem, mainly because of its cyclical and transgenerational character. In the health area, such actions can be carried out in Primary Health Care (PHC), such as in the FHS, which has as a pillar the development of strategic actions for preventing and coping with problems^(^ 36 ^)^.

For nursing in particular, studies in Canada and Australia have highlighted the salience of this qualification, based on a comprehensive curricular training on the subject, favoring the recognition of cases, welcoming and accompanying women during the decision process to break the violent cycle and also afterwards^(^ 37 ^-^ 38 ^)^, in order to prevent women from resuming the relationship in the same disrespectful patterns.

## Conclusion

Through the experiences of women in lawsuits because of marital violence, the study allowed the emergence of the phenomenon “Experiencing conjugal violence as a progressive and cyclical process, with repercussions for health and implications for social relations.” Although findings in segmented form do not bring innovations about what is reported in the national and international literature, the study, by proposing a theoretical model representative of the phenomenon, articulates knowledge in the sense of enabling coping strategies.

In this perspective, the theoretical model of conjugal violence as a phenomenon that is expressed in a progressive and cyclical way, compromises the health and human development of women and their children and is related to the experience of domestic violence in childhood/adolescence, social construction of gender and the economic dependence of women on their spouses, points to the salience of institutional and social support, especially on the part of the family, as strategies that favor female empowerment, contributing to the end of a relationship of violence.

Considering that the study draws attention to conjugal violence as a recurrent problem in the life of women, making them and their children prone to illness, we must pay attention to these repercussions in the different spaces of care, whether legal, police or social or health-related, with a view to encouraging interventions for both groups. With regard to health environments, it is necessary to bring professionals closer to the issue of violence, especially to nursing professionals, due to their proximity to the community and their role in the management of care of FHS teams.

It is important to emphasize that, by providing care closer to this population, professionals working in PHC can provide care to women and children in situations of violence and direct them to different places in the care network, as well as being able to offer spaces for empowerment in the perspective of discussion groups. As for these spaces, it is worth mentioning that in addition to contributing to women’s emancipation concerning violence, its relevance is also due to the creation of new perspectives for a future free from this experience. In addition to this possibility of support, the contribution of family and friends as a social support for women experiencing violence is noteworthy. Therefore, women should also be encouraged to seek help such as this in order to achieve a break in the violent cycle.

In addition, understanding that the cycle of violence is not limited to the experiences of women, since children involved in this environment tend to reproduce such experiences in adulthood, educational spaces with this public are also urged. These spaces can also be articulated by ESF nurses, more specifically through the School Health Program, aiming to demystify the unequal relationship of gender involved in the violent process. It is important to note the limitation of the study regarding the transgenerationality of conjugal violence, given the need to deepen this theme in order to better understand how perpetuation of violence occurs throughout generations.
